# The test characteristics of head circumference measurements for pathology associated with head enlargement: a retrospective cohort study

**DOI:** 10.1186/1471-2431-12-9

**Published:** 2012-01-23

**Authors:** Carrie Daymont, Moira Zabel, Chris Feudtner, David M Rubin

**Affiliations:** 1 Department of Pediatrics and Child Health, The University of Manitoba, Winnipeg, Manitoba, Canada; 2 The Manitoba Institute of Child Health, Winnipeg, Manitoba, Canada; 3 Department of Pediatrics, The University of Pennsylvania, Philadelphia, Pennsylvania, USA; 4 Children's National Medical Center, Washington DC, USA; 5 Center for Clinical Epidemiology and Biostatistics, The University of Pennsylvania, Philadelphia, Pennsylvania, USA; 6 PolicyLab, The Children's Hospital of Philadelphia, Philadelphia, Pennsylvania, USA

## Abstract

**Background:**

The test characteristics of head circumference (HC) measurement percentile criteria for the identification of previously undetected pathology associated with head enlargement in primary care are unknown.

**Methods:**

Electronic patient records were reviewed to identify children age 3 days to 3 years with new diagnoses of intracranial expansive conditions (IEC) and metabolic and genetic conditions associated with macrocephaly (MGCM). We tested the following HC percentile threshold criteria: ever above the 95^th^, 97^th^, or 99.6^th ^percentile and ever crossing 2, 4, or 6 increasing major percentile lines. The Centers for Disease Control and World Health Organization growth curves were used, as well as the primary care network (PCN) curves previously derived from this cohort.

**Results:**

Among 74,428 subjects, 85 (0.11%) had a new diagnosis of IEC (n = 56) or MGCM (n = 29), and between these 2 groups, 24 received intervention. The 99.6^th ^percentile of the PCN curve was the only threshold with a PPV over 1% (PPV 1.8%); the sensitivity of this threshold was only 15%. Test characteristics for the 95th percentiles were: sensitivity (CDC: 46%; WHO: 55%; PCN: 40%), positive predictive value (PPV: CDC: 0.3%; WHO: 0.3%; PCN: 0.4%), and likelihood ratios positive (LR+: CDC: 2.8; WHO: 2.2; PCN: 3.9). Test characteristics for the 97th percentiles were: sensitivity (CDC: 40%; WHO: 48%; PCN: 34%), PPV (CDC: 0.4%; WHO: 0.3%; PCN: 0.6%), and LR+ (CDC: 3.6; WHO: 2.7; PCN: 5.6). Test characteristics for crossing 2 increasing major percentile lines were: sensitivity (CDC: 60%; WHO: 40%; PCN: 31%), PPV (CDC: 0.2%; WHO: 0.1%; PCN: 0.2%), and LR+ (CDC: 1.3; WHO: 1.1; PCN: 1.5).

**Conclusions:**

Commonly used HC percentile thresholds had low sensitivity and low positive predictive value for diagnosing new pathology associated with head enlargement in children in a primary care network.

## Background

Head circumference (HC) measurements are routinely performed at well-child visits in infants and young children. Despite the frequency with which these measurements are performed, little is known about how primary care physicians should use these measurements to distinguish sick from healthy children.

Macrocephaly, or an abnormally large head, is commonly defined as a head circumference above the 95^th ^percentile (corresponding in normally distributed HC values to 1.64 standard deviations from the mean of gender and age-specific controls) in the United States. This value was initially based on the inability to accurately determine more extreme percentiles in early growth curves [1]. Recommendations have also been made to use more extreme percentiles as a threshold for increased concern, such as the 97^th ^percentile proposed by the World Health Organization (WHO) [2] or the 98^th ^or 99.6^th ^percentile proposed for use in the United Kingdom [1,3]. National guidelines in Norway make use of another threshold, namely that a child whose head circumference has crossed two increasing major percentile lines should receive further evaluation [4]. A recent study using country-specific growth curves in Norway reported that this criterion had a sensitivity of 46% for intracranial expansive conditions (IEC) but did not provide information regarding specificity or predictive values [4].

Numerous pathologic conditions may cause an increased head size, including IEC such as hydrocephalus and chronic subdural hematomas, and metabolic and genetic conditions that may cause macrocephaly (MGCM), such as glutaric aciduria and Fragile X syndrome. The ability of these thresholds to accurately identify children with previously undiagnosed IEC and MGCM has not been evaluated.

We therefore conducted a retrospective cohort study to evaluate the performance of various threshold criteria for the identification of children with new diagnoses of IEC or MGCM in a primary care population receiving routine head circumference measurements.

## Methods

### Subjects and Data Sources

Electronic records of children who received care in a large primary care network associated with a tertiary care children's hospital were evaluated retrospectively. HC measurements are routinely performed at well child visits until three years of age in the network.

All subjects were born before 31 January 2008 and had at least one HC recorded in the electronic medical record before 31 January 2009 while they were between 3 days and 3 years of age. The HC measurements for these children had previously been used to create new HC growth curves [5]. Subjects with known birth weight less than 1500 grams or gestational age below 33 weeks were excluded.

Although HC curves may also be used to monitor the head growth of children with known diagnoses, our goal in this study was to evaluate the performance of HC curves for the identification of children with previously undetected pathology. Therefore, subjects were excluded if they had evidence of neurosurgery or a diagnosis of pathology known to be associated with an abnormally large head size before the first HC for that subject was recorded in the electronic record, regardless of whether the HC percentile was high. Subjects with diagnoses associated with small head size before the first HC was recorded were also excluded in order to avoid downwardly skewing the HC distribution of the final sample. Subjects with diagnoses made on prenatal ultrasound, which is performed routinely in our population, were excluded.

### Measures

The primary outcome of interest was the new diagnosis before three years of age of IEC or MGCM. The following were included as IEC: hydrocephalus (enlarged, not merely prominent, ventricles without evidence of brain volume loss); chronic subdural hematoma (with or without associated hydrocephalus); cyst (> 1 cm, causing mass effect or hydrocephalus); brain tumor (> 1 cm, causing mass effect or hydrocephalus) [4]. The following were considered MGCM: overgrowth syndromes (including acromegaly, Beckwith-Weidemann, Simpson-Golabi-Behmel Sotos, and Weaver syndromes), Alexander disease, cranial dysplasia, Canavan disease, Fragile X syndrome, galactosemia, gangliosidosis (GM_1 _and GM_2_), glutaric aciduria (type I and D-2-hydroxyglutaric aciduria), hemimegalencephaly, histiocytosis X, hypoadrenocorticism, hypoparathyroidism, Jacobsen syndrome, MASA syndrome, megalencephalic leukodystrophy, metachromatic leukodystrophy, mucopolysaccharidoses, neonatal progeroid syndrome, neurocutaneous syndromes (including neurofibromatosis type I, macrocephaly-capillary malformation, and multiple others), Noonan syndrome (and cardiofaciocutaneous, Costello, and Leopard syndromes), Opitz-Kaveggia syndrome, Peters-plus syndrome, peroxisomal disorders, progeroid form of Ehlers-Danlos, PTEN hamartoma syndromes (including Bannayan-Riley-Rubalcava and Cowden syndromes), Rett syndrome/X-linked MECP2 neurodevelopmental disorder, Robinow syndrome, sebaceous nevus of Jaddassohn, and Schwachman-Bodian-Diamond syndrome. The receipt of intervention for IEC or MGCM, including surgery, medication, special diet, or social services referral, was a secondary outcome [6-8].

We performed a secondary analysis including benign enlargement of the subarachnoid spaces (BESS) in the outcome because the clinical significance of this condition is controversial. Although BESS, when diagnosed, is rarely treated and the fluid collections generally resolve without intervention, some studies have raised concerns about the possibility of an association with subdural hematoma and increased rates of developmental delay [9-17].

### Independent Variables

In addition to demographic characteristics, independent variables included the HC percentiles and z-scores as determined by the Centers for Disease Control (CDC) [18] and World Health Organization (WHO) [2] growth curves as well as the primary care network (PCN) [5] curves derived from this cohort. The determination of HC z-scores and percentiles has been described previously. Efforts had previously been made to remove erroneous measurements [5]. During this evaluation we detected and excluded 3,439 additional measurements that were likely to be erroneous (1.3% of all measurements), primarily by identifying measurement pairs representing a decrease in HC.

### Data Abstraction

Demographic data, visit and billing codes, and HC were obtained on all subjects between the beginning of electronic record collection at that practice and 31 January 2009.

In order to identify subjects with IEC or MGCM, subjects with any of the following indicators in the clinical databases were evaluated with chart review: an outpatient diagnostic code for pathology that can cause abnormal head size; an order or result for neuroimaging; a referral to or evaluation by a relevant specialist; chromosome or genome analysis; or billing or diagnostic codes for neurosurgery. Subjects whose only indicator was an evaluation that occurred after the third birthday were not evaluated further. Chart review was limited to neuroimaging results that did not contain identifying information when possible.

Because practices in the network began using the electronic medical record at variable times, and because we evaluated children born as late as one year before our data collection stop-date, we had variable amounts of information on our subjects. To assess whether inclusion of subjects with incomplete data affected our results, we performed a sensitivity analysis restricted to subjects whose first recorded HC was before 1 month of age and whose last recorded HC was after 24 months of age.

### Data Analysis

All analyses were performed using Stata 11.2. Test characteristics for thresholds of the 95^th^, 97^th^, and 99.6^th ^percentiles were evaluated; a subject with any HC-for-age percentile above the threshold criterion was considered to be test-positive. The threshold criterion of crossing 2 increasing major percentile lines (MPL: the 5^th^, 10^th^, 25^th^, 50^th^, 75^th^, 90^th^, and 95^th ^percentile lines) was evaluated; for analytic thoroughness, criteria of crossing 4 and 6 increasing MPL were also evaluated. To determine the number of increasing MPL crossed, each subject's highest head circumference-for-age percentile was compared with his or her first percentile.

The sensitivity, specificity, and positive and negative predictive values, likelihood ratios, number needed to test, and number needed to screen for these thresholds for identifying a) all subjects with IEC or MGCM and b) subjects with IEC or MGCM who received intervention were determined.

The study was reviewed and approved by the Institutional Review Board of the Children's Hospital of Philadelphia.

## Results

We assessed 75,412 potentially eligible subjects. Of these, 984 were excluded because of evidence of a pre-existing diagnosis of an excluding condition before their first electronically recorded HC. Of the excluded subjects, 142 (14%) had a maximum HC over the 95^th ^PCN percentile, and 158 (16%) had a maximum HC under the 5^th ^percentile. There were 404,817 head circumference measurements on 74,428 remaining subjects (Table 1).

**Table 1:** Demographic characteristics of included subjects.

Sex	
Male	51%
Race	
White	50%
Black	33%
Asian	3%
Other	14%
Ethnicity	
Hispanic	3%
Median number HC measurements	5
Percent with > 1 HC measurement	85%
Median age first HC measurement (months)	1.2
Median age last HC measurement (months)	24.1

HC (head circumference)

### Identification of Subjects with Pathology

**Figure 1:**
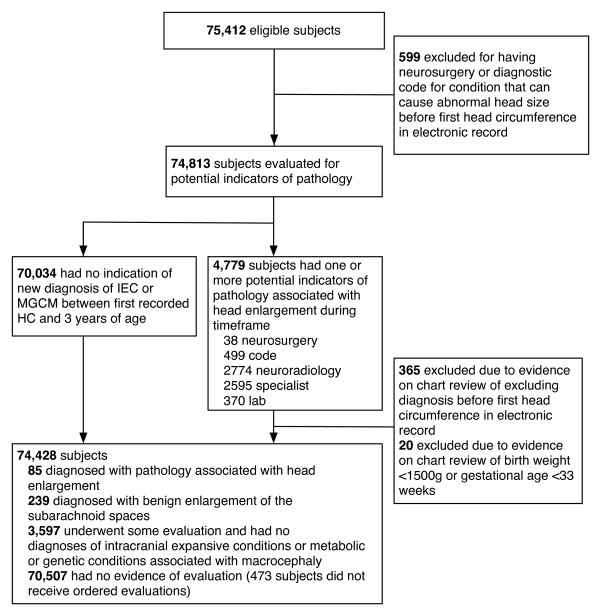
**Flowchart Describing Identification of Subjects with Outcome**. IEC (intracranial expansive condition), MGCM (metabolic and genetic conditions associated with macrocephaly).

Eighty-five subjects were found to have new diagnoses of pathology before three years of age (Figure 1). Of the 85 subjects with IEC or MGCM, 43 subjects had no diagnostic or surgery code and were identified because of the presence of neuroradiology orders or results, or specialist referrals or evaluations.

### Description of Diagnoses and Outcomes

Of the 85 subjects with the outcome, 56 had IEC: hydrocephalus (n = 24), chronic subdural hematoma (n = 15), cyst (n = 8), and tumor (n = 9). Twenty-nine had MGCM: neurofibromatosis (n = 8), tuberous sclerosis (n = 5), Beckwith-Wiedemann (n = 4), and 1 or 2 subjects each with the following diagnoses: glutaric aciduria type I, Sturge-Weber syndrome, Sotos syndrome, Fragile X syndrome, Noonan syndrome, Leopard syndrome, Bannayan-Riley-Ruvalcaba syndrome, hemimegalencephaly, X-linked MR associated with MECP2 duplication, and diffuse thickening of the skull with no known syndrome. None of the children with conditions classified as MGCM also had lesions large enough to be considered IEC.

There were 24 subjects who received specific intervention for pathology: 18 underwent surgery, 5 additional subjects did not receive surgery but were referred to social services because of concern for non-accidental trauma, and one was prescribed a special diet. Other subjects received variable degrees of further follow-up and evaluation, ranging from no follow-up for three subjects to multiple specialty evaluations and further neuroimaging.

### Cumulative Incidence

New diagnoses of IEC or MGCM were found in 0.11% (85/74,428) of the entire study population, with 0.03% (24/74,428) who had pathology with subsequent intervention. The age at diagnosis ranged from 3 days to 1075 days (median, 200 days). Eight subjects were diagnosed before 1 month; eight were diagnosed after 24 months.

### Head circumference characteristics of subjects with IEC or MGCM

**Figure 2:**
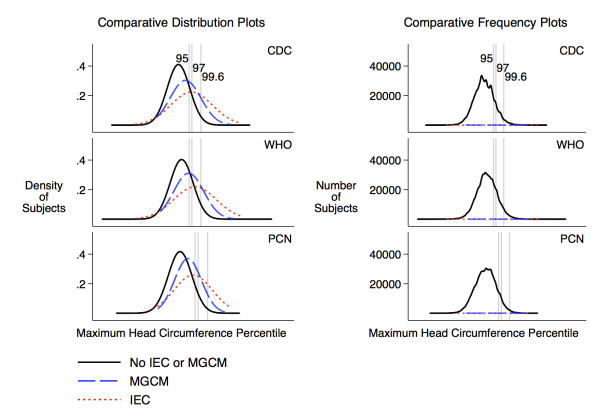
**Distribution of maximum head circumference percentiles by outcome**. The gray lines indicate the location of the 95^th^, 97^th^, and 99.6^th ^percentiles on the x-axis, which is scaled by z-score. The comparative distribution plots compare the distributions without regard to the number of subjects in each group. The comparative frequency plots (implemented using kernel density estimators) are scaled according to the number of subjects in each group (n = 73,343 for no IEC or MGCM, n = 29 for MGCM, n = 56 for IEC). The fact that the comparative frequency plots for subjects with pathology are flat reflects the small number of children in these categories compared to the number of children without pathology at most percentiles.

Subjects with IEC or MGCM had a wide range of head sizes, including some with HC below the 1^st ^percentile. The distributions of maximum HC percentile for subjects with pathology were different from the distribution for subjects without known pathology, but with a large amount of overlap (Figure 2).

### Test characteristics

The sensitivity, specificity, positive predictive value, positive and negative likelihood ratios, number needed to screen and number needed to test varied by threshold and curve source (Tables 2 and 3). The negative predictive value was 99.9% for each threshold. The threshold of crossing 6 major percentiles identified 490 (CDC), 556 (WHO) and 130 (PCN) children, but none of these subjects had pathology. Almost all of these children had a corresponding increase in weight and length z-scores of similar magnitude.

**Table 2:** Test Characteristics of Selected HC Thresholds for Diagnosis of Children with IEC or MGCM

A	B	C	D	E	G	H	I	K	L	M	N
**Threshold**	**Number in source population**	**Number diagnosed with IEC or MGCM**	**Number above threshold**	**Number above threshold with IEC or MGCM**	**Sensitivity** **E/C**	**Specificity** **(B-C-(D-E))/(B-C)**	**Positive predictive value** **E/D**	**Likelihood ratio positive** **G/(1-H)**	**Likelihood ratio negative** **(1-G)/H**	**Number Needed to Screen** **B/E**	**Number Needed to Test** **D/E**

**Above CDC 95th**	74,428	85	12,325	39	46%	83%	0.3%	2.8	0.6	1,908	316
**Above WHO 95th**	74,428	85	18,528	47	55%	75%	0.3%	2.2	0.6	1,584	394
**Above PCN 95th**	74,428	85	7,694	34	40%	90%	0.4%	3.9	0.7	2,189	226
**Above CDC 97th**	74,428	85	8,373	34	40%	89%	0.4%	3.6	0.7	2,189	246
**Above WHO 97th**	74,428	85	13,275	41	48%	82%	0.3%	2.7	0.6	1,815	324
**Above PCN 97th**	74,428	85	4,532	29	34%	94%	0.6%	5.6	0.7	2,566	156
**Above CDC 99.6th**	74,428	85	2,030	20	24%	97%	1.0%	8.7	0.8	3,721	102
**Above WHO 99.6th**	74,428	85	3,438	25	29%	95%	0.7%	6.4	0.7	2,977	138
**Above PCN 99.6th**	74,428	85	711	13	15%	99%	1.8%	16.3	0.9	5,725	55
**Crossed 2 IMPL-CDC**	64,015	83	29,206	50	60%	54%	0.2%	1.3	0.7	1,280	584
**Crossed 2 IMPL-WHO**	64,015	83	22,462	33	40%	65%	0.1%	1.1	0.9	1,940	681
**Crossed 2 IMPL-PCN**	64,015	83	13,831	26	31%	78%	0.2%	1.5	0.9	2,462	532
**Crossed 4 IMPL-CDC**	64,015	83	5,727	13	16%	91%	0.2%	1.8	0.9	4,924	441
**Crossed 4 IMPL-WHO**	64,015	83	4,372	7	8%	93%	0.2%	1.2	1.0	9,145	625
**Crossed 4 IMPL-PCN**	64,015	83	1,703	6	7%	97%	0.4%	2.7	1.0	10,669	284

IEC (intracranial expansive condition); MGCM (metabolic or genetic condition associated with macrocephaly); CDC (Centers for Disease Control head circumference growth curves); WHO (World Health Organization head circumference growth curves); PCN (primary care network head circumference growth curves); IMPL (multiple percentile lines). The negative predictive value (C-(D-E))/(C-D) was 99.9% for all thresholds. No subjects with the outcome crossed 6 increasing MPL, so rows for that outcome were not included. Point estimates and 95% confidence intervals are presented for the thresholds with the highest and lowest sensitivity and highest positive predictive value. The sensitivity of crossing 2 IMPL on the CDC curve for detecting children with IEC or MGCM who received intervention was 78% (95% CI: 56%, 93%). The sensitivity of crossing 4 IMPL on the PCN curve for detecting children with IEC or MGCM was 7% (95% CI: 3%, 15%). The positive predictive value of ever being above the 99.6^th ^percentile of the PCN curve for detecting children with IEC or MGCM was 1.8% (95% CI: 1.0%, 3.1%).

**Table 3:** Test Characteristics of Selected HC Thresholds for Diagnosis of Children with IEC or MGCM Requiring Intervention

A	B	C	D	E	G	H	I	K	L	M	N
**Threshold**	**Number in source population**	**Number diagnosed with IEC or MGCM requiring intervention**	**Number above threshold**	**Number above threshold with IEC or MGCM requiring intervention**	**Sensitivity** **E/C**	**Specificity** **(B-C-(D-E))/(B-C)**	**Positive predictive value** **E/D**	**Likelihood ratio positive** **G/(1-H)**	**Likelihood ratio negative** **(1-G)/H**	**Number Needed to Screen** **B/E**	**Number Needed to Test** **D/E**

**Above CDC 95th**	74,428	24	12,325	11	46%	83%	0.1%	2.8	0.6	6,766	1,120
**Above WHO 95th**	74,428	24	18,528	14	58%	75%	0.1%	2.3	0.6	5,316	1,323
**Above PCN 95th**	74,428	24	7,694	9	38%	90%	0.1%	3.6	0.7	8,270	855
**Above CDC 97th**	74,428	24	8,373	9	38%	89%	0.1%	3.3	0.7	8,270	930
**Above WHO 97th**	74,428	24	13,275	12	50%	82%	0.1%	2.8	0.6	6,202	1,106
**Above PCN 97th**	74,428	24	4,532	7	29%	94%	0.2%	4.8	0.8	10,633	647
**Above CDC 99.6th**	74,428	24	2,030	6	25%	97%	0.3%	9.2	0.8	12,405	338
**Above WHO 99.6th**	74,428	24	3,438	6	25%	95%	0.2%	5.4	0.8	12,405	573
**Above PCN 99.6th**	74,428	24	711	5	21%	99%	0.7%	22.0	0.8	14,886	142
**Crossed 2 IMPL-CDC**	64,015	21	29,206	18	78%	54%	0.1%	1.7	0.4	3,566	1,623
**Crossed 2 IMPL-WHO**	64,015	21	22,462	10	43%	65%	< 0.1%	1.2	0.9	6,402	2,246
**Crossed 2 IMPL-PCN**	64,015	21	13,831	9	39%	78%	0.1%	1.8	0.8	7,113	1,537
**Crossed 4 IMPL-CDC**	64,015	21	5,727	4	17%	91%	< 0.1%	1.9	0.9	16,004	1,432
**Crossed 4 IMPL-WHO**	64,015	21	4,372	2	9%	93%	0.1%	1.3	1.0	32,008	2,186
**Crossed 4 IMPL-PCN**	64,015	21	1,703	2	9%	97%	0.2%	3.3	0.9	32,008	852

IEC (intracranial expansive condition); MGCM (metabolic or genetic condition associated with macrocephaly); CDC (Centers for Disease Control head circumference growth curves); WHO (World Health Organization head circumference growth curves); PCN (primary care network head circumference growth curves); IMPL (increasing multiple percentile lines). The negative predictive value (C-(D-E))/(C-D) was 99.9% for all thresholds. No subjects with the outcome crossed 6 IMPL, so rows for that outcome were not included.

Crossing 2 increasing major percentile lines had the highest sensitivity but lowest positive predictive value, 0.1%-0.2% (diagnosis) and < 0.1%-0.1% (intervention). The only threshold with a number needed to test less than 100 for diagnosis of any new pathology was the 99.6^th ^percentile of the CDC curve (NNT = 55). The 99.6^th ^percentile of the PCN curve also had the highest likelihood ratio positive at 16.3 (diagnosis) and 22.0 (intervention), but had low sensitivity (15% diagnosis, 21% intervention).

The sensitivity analysis restricted to those 15,712 children with at least one evaluable HC recorded before 1 month and one after 24 months of age showed similar test characteristics. The cumulative incidence (0.19%) and positive predictive values for diagnosis for the 99.6^th ^percentiles were somewhat higher (CDC 1.5%, WHO 0.9%, PCN 3.4%), but the sensitivity of these criteria were low (CDC 27%, WHO 27%, PCN 23%).

When the 239 subjects diagnosed with BESS were included in the outcome (Table 4), the sensitivities (17%-75%), positive predictive values (0.7% - 9.7%) and likelihood ratios positive (1.4-24.6) were higher than for IEC and MGCM alone.

**Table 4:** Test Characteristics of Selected HC Percentile Thresholds for Diagnosing Children with IEC, MGCM, or BESS

A	B	C	D	E	G	H	I	K	L	M	N
**Threshold**	**Number in source population**	**Number diagnosed with IEC, MGCM, or BESS**	**Number above threshold**	**Number above threshold with IEC, MGCM, or BESS**	**Sensitivity** **E/C**	**Specificity** **(B-C-(D-E))/(B-C)**	**Positive predictive value** **E/D**	**Likelihood ratio positive** **G/(1-H)**	**Likelihood ratio negative** **(1-G)/H**	**Number Needed to Screen** **B/E**	**Number Needed to Test** **D/E**

**Above CDC 95th**	74,428	324	12,325	221	68%	84%	1.8%	4.2	0.4	337	56
**Above WHO 95th**	74,428	324	18,528	242	75%	75%	1.3%	3.0	0.3	308	77
**Above PCN 95th**	74,428	324	7,694	193	60%	90%	2.5%	5.9	0.4	386	40
**Above CDC 97th**	74,428	324	8,373	203	63%	89%	2.4%	5.7	0.4	367	41
**Above WHO 97th**	74,428	324	13,275	225	69%	82%	1.7%	3.9	0.4	331	59
**Above PCN 97th**	74,428	324	4,532	167	52%	94%	3.7%	8.8	0.5	446	27
**Above CDC 99.6th**	74,428	324	2,030	129	40%	97%	6.4%	15.5	0.6	577	16
**Above WHO 99.6th**	74,428	324	3,438	155	48%	96%	4.5%	10.8	0.5	480	22
**Above PCN 99.6th**	74,428	324	711	69	21%	99%	9.7%	24.6	0.8	1,079	10
**Crossed 2 IMPL-CDC**	64,015	321	29,206	223	69%	54%	0.8%	1.5	0.6	287	131
**Crossed 2 IMPL-WHO**	64,015	321	22,462	162	50%	65%	0.7%	1.4	0.8	395	139
**Crossed 2 IMPL-PCN**	64,015	321	13,831	156	49%	79%	1.1%	2.3	0.7	410	89
**Crossed 4 IMPL-CDC**	64,015	321	5,727	103	32%	91%	1.8%	3.6	0.7	622	56
**Crossed 4 IMPL-WHO**	64,015	321	4,372	66	21%	93%	1.5%	3.0	0.9	970	66
**Crossed 4 IMPL-PCN**	64,015	321	1,703	55	17%	97%	3.3%	6.7	0.8	1,143	30
**Crossed 6 IMPL-CDC**	64,015	321	490	17	5%	99%	3.5%	7.1	1.0	3,766	29
**Crossed 6 IMPL-WHO**	64,015	321	556	17	5%	99%	3.1%	6.3	1.0	3,766	33
**Crossed 6 IMPL-PCN**	64,015	321	130	10	3%	> 99%	7.7%	16.5	1.0	6,402	13

IEC (intracranial expansive condition); MGCM (metabolic or genetic condition associated with macrocephaly); BESS (benign enlargement of the subarachnoid spaces); CDC (Centers for Disease Control head circumference growth curves); WHO (World Health Organization head circumference growth curves); PCN (primary care network head circumference growth curves); IMPL (increasing multiple percentile lines). The negative predictive value (C-(D-E))/(C-D) was 99.9% for all thresholds.

### Description of subjects with pathology below the CDC 95^th ^percentile

There were 46 subjects with pathology with IEC or MGCM whose head circumference was never above the CDC 95^th ^percentile, 13 of whom received intervention. The 25 subjects with IEC (7 with hydrocephalus, 5 with cysts, 9 with subdural hematomas, and 4 with tumors) were diagnosed because of increasing HC percentile, acute altered mental status that led to the diagnosis of underlying chronic subdural hematomas, or other neurologic signs. The 21 subjects with MGCM were primarily diagnosed because of characteristic signs unrelated to head size, such as macroglossia or café-au-lait spots.

## Discussion

The prevalence of undiagnosed IEC and MGCM in our primary care population was lower than the overall prevalence of these conditions. Many children with IEC and MGCM are identified before their first primary care visit through prenatal ultrasound, newborn metabolic screening, or evaluation in the nursery or neonatal intensive care unit. Importantly, our findings are therefore not applicable to newborns in the nursery or neonatal intensive care unit. One case series suggests that children born with a high HC percentile have a higher risk of significant pathology than children who develop a high HC percentile later [19].

Many of the subjects with IEC or MGCM, including subjects with hydrocephalus, had typical or even small head sizes. One explanation for the large number of children with pathology who had small or typical head sizes is that some conditions associated with head enlargement will not always cause any increase in head size. For example, neurofibromatosis is often associated with increased head size but has a variable phenotype and may not always cause increased head size. Furthermore, HC does not account for all variation in head size [20]: some conditions may cause an increase in intracranial volume primarily by increasing the height of the intracranial space, but not the occipital-frontal circumference. A third explanation involves the wide variation in normal HC for each age and sex: for many of the subjects with pathology but without a large HC-for-age, the pathologic condition may have caused an increase in head size compared to the smaller head size that child would have otherwise had, but this increase may not have been sufficient to raise the child's HC above the recommended percentile cutoffs.

Future research must focus on determining the elements of the history and physical examination that are most useful for the early identification of IEC or MGCM, or for reducing the number of unnecessary diagnostic imaging evaluations among children with large HCs. Three methods seem to have the most potential for obtaining more information from the HC itself. First, clinicians could evaluate the rate of change in HC over time, in a manner more precise than measuring the number of crossed major percentile lines, such as with growth velocity curves. Unfortunately, accurately evaluating growth velocity is fraught with difficulty since comparing two measurements compounds the effects of measurement error, and since head growth occurs in a variable sequence of relatively slow and fast periods [21-24]. Second, the association between head circumference and other growth parameters, such as height and weight, may provide valuable clinical information [25-27]. Third, further study of the information provided by the head circumference of parents and other relatives could be important in evaluating the significance of a given child's large HC.

Autism was not included in the outcome definition. Autism has been found to be associated with enlarged HC in some clinical samples [28,29], but other studies, including a longitudinal evaluation of a large community-based sample, have not found an independent association [30,31]. We do not believe that identifying children who may be at minimally increased risk of autism has been, or should be, one of the goals of routine HC measurements.

We included BESS in a secondary analysis rather than the primary analysis because we do not believe that it is important to identify all children with BESS. It is not clear that BESS is at all pathological, and BESS is not treated in most centers. Even if BESS is shown to be associated with developmental delays which are not detected by routine screening and for which detection is beneficial, it does not seem necessary to expose children to radiation or sedation in order to determine which children should receive extra developmental testing. BESS may be associated with an increased risk of subdural hematoma, but we are not aware of any methods to prospectively prevent those subdural hematomas beyond measures that would be considered proper care for any infant.

The most important limitation to our study is the variable follow-up time. A sensitivity analysis restricted to those children for whom electronic information was available before 1 and after 24 months of age did not change the overall conclusion. We also relied upon medical records to identify children with pathology. Although we believe most children, especially those with IEC, would have been identified, some children may not have been diagnosed by three years of age. Furthermore, despite efforts to exclude erroneous measurements, some were certainly still included.

The strengths of our study include extensive efforts to accurately identify all children with new diagnoses of pathology. Evaluation of administrative data alone would have caused a large degree of misclassification.

## Conclusions

The majority of children with large heads in our primary care population, even those with a HC larger than three standard deviations from the median or crossing multiple increasing major percentile lines, did not have evidence of a diagnosis of IEC or MGCM. Children with a very high HC percentile have an increased risk for pathology compared to other children, as indicated by a modestly elevated positive likelihood ratio. Their absolute risk of pathology, however, is small because of the low baseline prevalence of undiagnosed pathology in this primary care population, as illustrated by the relative frequency plots. Furthermore, a substantial proportion of patients with IEC or MGCM had HC percentiles below the tested thresholds. Our findings reinforce that physicians should not be reassured by a normal, or even low, HC percentile if there are other signs or symptoms suggestive of conditions associated with an increased frequency of macrocephaly.

Our findings highlight the difficulty primary care physicians face when they try to identify asymptomatic children with early-stage intracranial pathology while minimizing unnecessary investigations and worry to parents. Further research in other populations and, ideally, prospective cohort studies are necessary to provide physicians with a stronger evidence base regarding the use of these frequently performed measurements.

## Competing interests

The authors declare that they have no competing interests.

## Authors' contributions

CD conceived the study, participated in its design and data collection, performed the statistical analysis, and drafted the results, method, and discussion. MZ participated in data collection and drafted the introduction. CF and DR conceived the study, participated in its design, and helped to draft and critically revise the manuscript. All authors read and approved the final manuscript.

## Pre-publication history

The pre-publication history for this paper can be accessed here:

http://www.biomedcentral.com/1471-2431/12/9/prepub
